# Christianity and reverential naturalism engage a world in peril: A dialogue between disciplines

**DOI:** 10.1177/00377686241276385

**Published:** 2024-09-20

**Authors:** Paul BRAMADAT, John THATAMANIL

**Affiliations:** University of Victoria, Canada; Union Theological Seminary, USA

**Keywords:** Christianity, dialogue, naturalism, process theology, sociology, christianisme, dialogue, naturalisme, sociologie, théologie du processus

## Abstract

Christian theologians and ostensibly secular sociologists of religion rely on different resources to respond to personal, local, or global problems. The environmental crises we see around the world reflect a strict hierarchy between human beings and the natural world. In both the theological and social scientific arenas of the last few decades, however, we see an ‘animal turn’ that exposes the hubris of anthropocentrism and creates opportunities for new ways of writing and teaching about the natural environment. Using the Pacific Northwest’s ‘reverential naturalism’ as a touchstone, the authors reflect on the extent to which their respective fields prepare secular scholars and theologians to address the crises all animals – including humans – now face. Using a dialogue format, they explore whether theological and social scientific regimes of truth and knowledge are incommensurable. What might this mean for the region and the two fields out of which they authors emerge?

## Résumé

Les théologiens chrétiens et les sociologues des religions apparemment laïcs s’appuient sur différentes ressources pour répondre à des problèmes personnels, locaux ou mondiaux. Les crises environnementales que nous observons dans le monde reflètent une hiérarchie stricte entre les êtres humains et le monde naturel. Cependant, dans les domaines théologique et social des dernières décennies, nous assistons à un « tournant animal » qui expose l’hubris de l’anthropocentrisme et ouvre à de nouvelles manières d’écrire sur et d’enseigner l’environnement naturel. En s’appuyant sur le « naturalisme révérencieux » du nord-ouest du Pacifique comme point de référence, les auteurs réfléchissent à la façon dont leurs domaines respectifs préparent les chercheurs laïcs et les théologiens à affronter les crises auxquelles tous les animaux – y compris les humains – sont désormais confrontés. Par le biais d’un dialogue, ils se demandent si les régimes de vérité et de connaissance de la théologie et des sciences sociales sont incommensurables. Que cela pourrait-il signifier pour la région et les deux domaines dont les auteurs sont issus ?

## Introduction

This special issue addresses themes that could hardly be more abstract and yet also very mundane. Surely, the now ubiquitous climate crises we see around us demand our attention. We, as a single species but also as just one kind of resident of a complex biosphere, find ourselves at what one would have to call an existential moment. So the stakes are high and the changes are fundamental. And yet, one way to imagine a new paradigm that might forestall the truly catastrophic outcomes of increasingly volatile climate change, is through a fundamentally new approach to the other animals with whom we share this planet – those curled up on our couches, those on whose labour or flesh we rely, those we admire from our cars, those sitting across from us on the subway, and those whose extinction we would register as a deep loss.

Of course, one of the obstacles to discussing these distantly global, and yet also very immediate challenges, is that the fields and disciplines that shape us sometimes seem to make it difficult to find a common language, not to mention shared normative or methodological orientations. We – Paul Bramadat, a religious studies scholar, and John Thatamanil, a theologian – initially thought that while we, as friends, share a personal affinity and sense of the peril we all face, perhaps our different intellectual moorings might mean we would find very little professional common ground from which to reflect on the core question the editors have set for all contributors. And so, we decided to co-author this article as a dialogue between ourselves, as representatives, so to speak, of our respective fields. While we might well find that our approaches – as such and *vis à vis* thinking about the animal turn and its emergence at a time of climate emergency – are incommensurable, it might also be the case that the differences between the worlds in which we work are less categorical than they might appear.

Before we engage one another, consider Peter Berger’s (1967) argument about why the study of society is a distinctively human enterprise:

Human being is externalizing in its essence and from the beginning… Homo sapiens occupies a peculiar position in the animal kingdom. This peculiarity manifests itself in man’s relationship both to his own body and to the world. Unlike the other higher mammals, who are born with an essentially completed organism, man is curiously ‘unfinished’ at birth (…). The non-human animal enters the world with highly specialized and firmly directed drives. As a result, it lives in a world that is more or less completely determined by its instinctual structure (…). Man’s world is imperfectly programmed by his own constitution. It is an open world. That is, it is a world that must be fashioned by man’s own activity. Compared with the other higher mammals, man thus has a double relationship to the world. Like the other mammals, man is in a world that antedates his appearance. But unlike the other mammals, this world is not simply given, prefabricated for him. Man must make a world for himself (Berger, 1967: 11–13).

As we can see from Berger’s classic 1967 argument for the sociological approach to religion, the animal is written out from the beginning of the field. The human is defined in terms of its difference from and one might say exceptionality in its relationship to the natural world understood more broadly. On the one hand, this would seem to make the human animal unique in the sense that only we are capable of seeing our social and material world as literally artificial. Arguably, this would put Berger’s philosophical anthropology in line with others who see the human animal as *sui generis*, or in some sense categorically different and destined to do more than accept the world as we find it.

One might quibble with Berger. First, one could argue that many life-forms – from beavers and bees to fungi and ferns – have impacts on their surroundings that reflect their survival needs. Second, one could observe that while humans may uniquely possess the capacity to consider and reinvent the world in which they find themselves, and while much that is worthwhile in our long history emerges out of that capacity, in truth, a great many of us act strenuously to disavow this capacity and prefer to think about our social and material surroundings as little as possible. Yes, humans by definition change their worlds, and such change presupposes an awareness of one’s freedom to do so (here, Berger is right), but we often overstate both our exclusive capacity to alter our worlds and, most importantly, the enthusiasm with which people approach this capacity.

On the other hand, there is promise in Berger’s exercise in situating the human being as a peculiar sort of animal whose distinctive biology leaves *this* animal unfinished until completed by culture. Berger must be credited for both affirming human animality and situating that animality within evolutionary history. These are promising openings for any sociology that aspires to take animality seriously inasmuch as Berger recognizes the human being as an animal even if he frames the human as that exceptional animal capable of creating, recreating, and considering new artifices (which is to say, culture).

One hardly needs to look hard for analogous canonical texts and figures within the Christian tradition that render animals invisible, insignificant, or inscrutable. There are exceptions, but arguably both Christian theology and the sociology of religion have been anthropocentric from their beginnings. However, as Halafoff and Beaman note in their introduction to this special issue, many things have changed in our fields, and one of the most interesting shifts in the last two decades at least has been the so-called ‘animal turn’, or the effort to broaden our analyses to adopt a more inclusive definition of persons, beings, sentience, consciousness, souls, and society. We both applaud the turn towards these issues in our fields.

Looking at 1967 from the contemporary moment, we can see the hopeful beginnings of so many social changes from which many have benefitted and yet also so many other changes (e.g. deforestation, large scale agriculture, suburbanization) that have had devastating planetary consequences. This is not the place to point fingers at our governments, corporations, religious communities, or intellectual ancestors, for being ignorant of what many of us might now see more clearly. However, it seems both worthwhile and we hope also interesting, to wonder about our respective approaches to the current epoch. Indeed, it could be the case that specific places in our world – here, the Pacific Northwest is employed as an example – might have something to teach sociologists and theologians in other parts of the world about a way through some of the tensions we trace. Finally, it might well be that in a conversation between people moored in different sciences, we can find a common approach to addressing the frightful situations – climate change, political polarization, and populism – that have arguably inspired the animal or ecological turn we see in our respective fields.

We both agree that we stand at an unprecedented moment in human history and thus that the implications of maintaining the walls between our respective ‘secular-sociological’ and ‘theological’ orientations are, frankly, dire. As such, we ask readers of this journal who are accustomed to conventional scholarly approaches to indulge us a little. What follows is a critical dialogue between two colleagues and friends on the theme of this special issue. That conversation reflects our relationship and specific reading of the special issue’s problematic, but we hope there might be broader value in such an engagement.

It might be worthwhile for readers to know that each author is moored not just in different disciplinary but also ethno-religious harbours. In particular, Bramadat is of mixed-race descent, with an Indo-Trinidadian father and Anglo-Celtic prairie-born Canadian mother; he was raised as a Unitarian Universalist and now resonates mostly with a particular set of yogic philosophies and practices. Thatamanil is a 1.5 generation immigrant born in the South Indian state of Kerala who migrated to Brooklyn when he was 8 years old. His family belongs to the Mar Thoma Church, which traces its origins from the Apostle Thomas, who, according to tradition, came to India in 52 CE. This uprooting to the United States, together with his Christian background, prompted in Thatamanil a desire to know more about Indian religions. He works as a comparative theologian with special academic and spiritual interests in Buddhist and Hindu traditions of non-dualism. He is also an Anglican priest.

## Living well together with all forms of life: a social scientist’s reflection

In the call for papers for this special issue, contributors were encouraged to take up a question posed by Haraway and Derrida: How can we live well together with all forms of life?

At the outset, I [Paul Bramadat] should confess I have some discomfort with this question. First, the question asks me to assume that I might know how we can *do* something (live well), which is to say that I might have special knowledge of some technique or fact, and I might want to take it upon myself to explain or encourage others to do this thing (living well) I know how to do. In other words, this question is about not just knowing, but knowing-how, and especially knowing-how-to-do-something-others-should-do. Indeed, some of my research has been directly applied and ‘policy-relevant’, in that I have sometimes been asked to link my understanding of a phenomenon – say, youth radicalization or vaccine hesitancy – with practical suggestions about how policy-makers might respond more effectively and wisely to such things (Bramadat and Dawson, 2014; Bramadat et al., 2017). While the discomfort with ‘applied’ research has not left me, I have also learned a great deal from these projects – both about the phenomenon in question and also about the often very different pressures faced by policy makers on the one hand and scholars on the other.

Second, the Haraway/Derrida framing question asks me to share my know-how about living well *together*. What might it mean for a religious studies scholar or a sociologist of religion, to speak about living well together? Certainly, I can say something about how members of particular groups – for example, Canadian yoga practitioners, or vaccine hesitant parents – understand what it means to live within a particular community according to that group’s norms and values. I can say something about the obstacles they experience in their efforts to live as they choose. But should I say anything about living well as such? I am suspicious about such an objective for a scholar, or at least for the kind of scholar I want to be. Would a claim about living well not be, in the final analysis and perhaps from the beginning of the undertaking, a personal, idiosyncratic, and perhaps theological project? Of course, there may be room for a critical approach to religious phenomena to trace – in a non-dispassionate way – the ideologies that produce our groups and ideas (Mosurinjohn and Watts, 2021). That is possible, but subject also to the pitfalls one sees in more straightforwardly normative theological undertakings.

There is a lot of merit in the effort to be conscious of one’s own biases, and to suspend them when possible. According to the broader story animating the academy, this is the effort that makes one’s conclusions – here comes the magic word – scientifically credible. Nonetheless, the Haraway/Derrida question makes me cringe a little. John, I assume that you, as a theologian, find nothing untoward about being asked to say something about the ways one ought to live well together. After all, I take it to be your job to think about but then finally – since you are a scholar and a priest – to demonstrate how others might behave, think, and feel in ways consistent with the principles embedded within your tradition. For me, though, to say one does anything *well* is to speak relatively – one dances well relative to particular aesthetic norms, for example. Regardless of how one might *feel* about dancing, one dances *well* according to the dominant actions, habitus, and skills associated with a given dance form and the culture to which it is connected.

If ‘*how* can we’ resembles too closely the ‘best practices’ orientation of policy-relevant work (which is neither my main interest nor the key concern of scholarly work); and if ‘live well together’ requires me to pursue normative objectives, the scientific bona fides of which seem to be not self-evidently desirable; then perhaps the rest of the question that is, ‘(…) with all forms of life’, might lead me out of this thicket? Here, we reach a door in this argument, and the third of my concerns. By asking contributors to this special issue to ask how we can live well together *with all forms of life*, I take it Halafoff and Beaman are signalling that professional scholars might be (along with everyone else) at the beginning, or in the middle of, a categorically different historical epoch. That is, perhaps our species has entered the Anthropocene, a time in which human beings have become the preeminent shapers of the global environment and thus obligated to think anew about our relationships with all forms of life.

At this point, I might invoke the philosophical equivalent of Canada’s constitutional sky-hook, also known as the ‘notwithstanding clause’. In Canadian constitutional history, provinces are permitted to pursue policies *notwithstanding* the legal and ethical principles by which they have otherwise formally agreed to abide and that explicitly and philosophically seem to forbid the policy in question, only when they can argue that some supervening political or moral objective is at stake (Oliver et al., 2017).

This clause has done a great deal in Canadian history, and it might afford me an exit from the usual methodological atheism or agnosticism that is often assumed to be the *modus operandi* of the social scientist. It might be that the dark prospects of a ruined climate that will doom the lifeworlds in which all animals – including humans, of course – move, makes it not just possible, but urgent, for social scientists who see their labours as unfolding within an ostensibly secular regime of truth and knowledge, to provide advice regarding living well. This advice would, or should, be offered not just from the vantage point of an external adjudicator vis a vis some specific regime but as one being among trillions, deeply embedded in an eco-system upon which ‘all forms of life’ depend. I am inclined to think this is indeed an appropriate use of a scholarly notwithstanding clause. Nonetheless, writing something that is both scholarly and still advances a normative agenda for ‘all forms of life’, does lead to some anxiety.

## On disciplinary constraints: a theologian’s response

In his 2004 essay, Bruno Latour (2004) worries that climate change denialists use the same strategies to sabotage the science establishing anthropogenic climate change that he himself deployed in his work in science studies to situate scientific activity within the larger social matrix of its production. Writing about science denialists, Latour wonders,

Do you see why I am worried? I myself have spent some time in the past trying to show ‘the lack of scientific certainty’ inherent in the construction of facts. I too made it a ‘primary issue’. But I did not exactly aim at fooling the public by obscuring the certainty of a closed argument – or did I? After all, I have been accused of just that sin. Still, I’d like to believe that, on the contrary, I intended to emancipate the public from prematurely naturalized objectified facts. Was I foolishly mistaken? (Latour, 2004: 227)

I [John Thatamanil] do not intend to rehash this particular debate. My intention is more circumscribed: to flag a crucial moment in which social scientific scholarship came to wonder about its own purposes. *What is social science for?* Should the social scientist be rigorously committed to objectivity and direct critique in service to that goal? Or must they aim at some more capacious human or more-than-human goal such as ‘living well together?’ Latour’s openness to a reconsideration of the vocational objectives of social science was prompted both by worries about the post-truth turn and the climate crisis. Paul’s invocation of the Canadian notwithstanding clause takes its bearing from the latter and not so much the former worry about the cultural spillover of the corrosive acids of academic critique. He is willing to open himself to the urgent call of this thematic volume despite its normative framing because social science has no future if the human species itself has no future.

Paul intimates that theologians like me are likely to be less put off by the normative aspirations of this volume. Theology is, after all, unlike sociology of religion, an unabashedly normative enterprise. Paul, you are not wrong: a theologian without normative investments is as fantastical a creature as a bird without wings. Still, I find myself uneasy about a full-throated embrace of any characterization of my work as normative *as opposed to* scientific. Many theologians construe their work as a species of *wissenchaftlich* scholarship. That is, they strive to be self-aware and self-critical about their socioeconomic locations and political investments, and some seek to do theology within the constraints that come with a non-reductive scientific naturalism. Arguably, most forms of liberal theology, certainly process theology that follows in the wake of the British-American philosopher and mathematician, Alfred North Whitehead (1925), and the theology of Paul Tillich (1967) – the two streams of theology to which I am most indebted – refuse accounts of divine power that make divine interruption of natural causation possible. God either cannot or does not interrupt natural causal processes. God’s actions, therefore, must take place through worldly processes not by their circumvention. In these theological traditions, God is not an omnipotent being who can at His whim – the masculine form signals the associations of such theologies with patriarchy – break into the world’s ordered patterns whenever He so chooses. Embracing naturalisms means that many contemporary theologians accept that giving an account of *how* the natural world works is not their domain but of the various sciences. In this respect, liberal and progressive theologians work within university norms and so accept radio-carbon dating and other methods for establishing the age of the universe, embrace evolutionary theory unless some insist that it should be employed to endorse predatory social Darwinism, and the like.

Theologians resist these norms only when scientists slip into venturing metaphysical claims – that is when scientists claim that nature is nothing else and nothing more than what can be studied by the empirical sciences. Even a religiously sparse ‘reverential naturalism’ of Paul’s sort would and could not be rationally entertained on such scientistic grounds. What, after all, is there in nature that is worthy of reference if nature consists of nothing but bits of matter in motion?

In sum, most liberal or progressive theologians try to engage in theological labour that takes for granted Darwinian evolution, Big Bang cosmology (or steady state cosmology if the science should so warrant), and accounts of causation that do not permit arbitrary divine incursion. Theological method for such theologians is not of a wildly different stripe in its working assumptions than the assumptions that structure your work, Paul. Working as many of us do on both sides of the religious studies/theology divide, at least some theologians are all too aware of how much work it takes to render any normative claim plausible to fellow inmates of the ivory tower.

The very notion that there is in fact a stark divide between religious studies and theology is itself an artefact of a time in which religious studies scholars were (rightly) seeking to emancipate themselves from the constraints of Christian theology. But that emancipation has proved partial at best – as covert or uncritical assumptions about what ‘religion’ and ‘the religions’ were shaped not only by uninterrogated Christian norms but more seriously by conceptions of ‘religion’ emerging from the production of the secular versus religious bifurcation in European modernity. Thanks to the work of genealogists of religion and postcolonial theorists, both religious studies and theological scholars should now be far less willing to pretend that the secular-religious binary – which structures the disciplinary binary between religious studies (secular) and theology (religious) – is a discovery of natural kinds (Asad, 1993, 2003; Chidester, 2014; King, 2013; Mandair, 2009).

The provincial European production of this bifurcation and its inapplicability for the study of Indigenous traditions (and, in fact, most of human religious history) has become increasingly apparent in the wake of the work of Talal Asad and others. Put simply, there is no natural sphere of the secular that was always already waiting to be discovered and emancipated once the ‘religious’ has been distilled out and quarantined in private inward space. This invention and its global imposition through colonialism has been well studied by both religious studies scholars and theologians. What remains to be seen is whether religious studies scholars will realize the methodological implications of these discoveries for how they construe their own work as ‘secular’. How is the insistence that only secular methodologies properly count as ‘scholarly’ to escape the charge that such claims enfranchise some and disenfranchise others, particularly those who do not recognize the secular-religious binary to begin with?

If much progressive theological work strives to account for and work within contemporary academic knowledge regimes, even as we critique the supposed neutrality of ‘the secular’, I think it would be wise, Paul, not to overstate the divide between social scientists and at least some academic theologians. While you are right to suggest that I am not as constitutionally allergic to the normative question of ‘living well together’ as you are, you need not imagine all Christian theologians as folks who are ready at all times to let our normative freak flags fly. Theological knowledge are not wholly captive to regnant university discourses, but we too are formed by analogous scholarly practices and habitus as our social scientific colleagues. In sum, you and I are both subspecies of *homo academicus*, and the differences between us need not be exaggerated.

That said, Paul, you are right that we theologians need not be backed into a desperate corner and driven to invoke an emergency notwithstanding clause to acknowledge that our scholarship is driven by normative goals. Christian ecotheology has been a flourishing subdiscipline within theology at least since the work of process theologian John Cobb’s early groundbreaking work, *Is it Too Late? A Theology of Ecology* (1972). The pioneering works of Sallie McFague (1987) first triggered to theological reimagination by the Cold War threat of nuclear apocalypse, also turned organically to the planetary context necessary for flourishing human life. The specifically animal turn, of course, is more recent (Calarco, 2015; Johnson, 2013), but in this respect Christian theologians are not woefully behind social scientific colleagues. As Berger’s articulation of the fundamental distinction between humans and other animals suggests, there is no good reason to suppose that Christian theology is more plagued by benighted anthropocentrism than social science. Arguably, the foundations of contemporary sociology of knowledge and religion are at least as anthropocentric if not more so than some varieties of Christian theology.

Christian ecotheology has been preparing the way to make the animal turn for some time now. For example, Christian theologians from a variety of schools of philosophical theology reject the soul-body dichotomy and theological assumptions that human salvation is primarily a matter of liberating an immaterial human soul from a decaying and decadent flesh that is unimportant *sub specie aeternitatas*. Theologians such as Paul Tillich and the entire process theology community reject such soul-body dualisms and urge Christian reflection to regard ‘spirit’ as a way of speaking of human agential purposiveness rather than as a supernatural substance. By rejecting talk of soul as supernatural substance, theologians have long prepared for revaluing embodiment more generally. Although it is beyond the scope of this article, it is worth noting that one would be hard-pressed to find anything like a body-soul dualism in East Asian traditions. The nearest analogate to spirit would be the notion of chi/qi, but chi is best understood as material energy rather than as disembodied spirit. In some South Asian traditions, there is also a consciousness (*cit*) versus mind-body complex dualisms or spirit (*purusa*) versus material nature (*prakrti*) dualism, but it is worth noting that in both South and East Asian traditions, matter is not inert mechanism. In Indian traditions, including the Yogic traditions Paul studies, mind is classed on the matter side of consciousness/matter divide. In several of these traditions, in other words, matter is not reducible to inert and insensate atoms in motion.

The dismissal of depictions of matter as inert mechanism driven solely by external efficient causation is also a preparation for a richer view of nature within which a turn to animal life can be imagined. Tutored by a whole host of approaches that seek ‘enchantment without supernaturalism’, including non-mechanistic work in quantum physics and relativity, theologians have sought to combat reductive and mechanistic accounts of matter – accounts that make it easy to imagine animals as mere mechanism, particularly as they lack a supernatural soul (Griffin, 2000).

Also noteworthy are theologies of incarnation that stress not only that God became human but that in becoming human God became also becomes matter. Under the core theological principle, ‘what has not been assumed cannot be saved’, Christian theologians have long insisted – really from at least the fourth century – that in and through the incarnation, God has redeemed all matter, not just humanity. On this score, we can imagine incarnation as God’s becoming not just human but animal. The latter claim would be a rather obvious extension of classical accounts of divine incarnation.

Theologies of creation have also put in place a radical intimacy of divinity to nature and materiality that is a necessary precondition to any animal turn. Consider the argument by Kathryn Tanner (2004) and Robert Neville (1992) that an insistence on *creatio ex nihilo* means that God needs nothing whatsoever to create with. God’s creative work operates without intermediaries and so is intimately present to each and every creature. This claim of immediate presence deftly undercuts Great Chain of Being (GCOB) theology in which divine creativity is hierarchically mediated. In GCOB theologies, God is closer to creatures of higher rank which are nearer to divine than lower creatures whose diminished position in the hierarchy is marked by their greater materiality. By contrast, when God creates out of nothing, God is as close to any element in creation as to any other, animals included. To be emphatically clear, the rejection of GCOB theologies and its explicit avowal that some creatures, most especially humans, are closer to the divine than other creatures, namely animals, is an essential prerequisite for any meaningful animal turn.

Wary about the claim that God creates out of nothing by overwhelming creative fiat, other theologians such as feminist apophatic theologian Catherine Keller argue for *creatio ex profundis* – a creation out of the depths – in which these depths are not external to but rather internal to divinity. Such claims also remove any claim that divine presence must be mediated through a highly ramified GCOB, in which some beings have higher ontological status than others. On both these accounts of creation, God is no nearer to human beings than to any other creature, organic or inorganic. Keller has worked out the feminist and even ecofeminist implications of her process-inflected vision of creation out of the depths in a variety of books and essays throughout her distinguished career. Her position is a panentheism in which the world is in God and God in the world, but neither can be reduced to the other. Within such an imagination, *all* creatures without exception are part of the divine life and the divine life part of every creature. Incarnation is not an exceptional event that takes place only in one exceptional human, but rather an ongoing process in which all creatures, animals included, incarnate or rather ‘intercarnate’ divinity (Keller, 2017: 3).

Here, Keller is arguably in continuity with classical Christian theologians who in the early centuries argued that the incarnation was not just the Logos taking on humanity alone, but, on the contrary, because the human is a microcosm which contains every aspect of creation, incarnation might as well be regarded as God’s becoming an earthling or God’s becoming animal. While the implications for an animal turn were not rendered explicit in these early centuries, they can and ought to be today. When the Word assumes flesh, it assumes all of creation, animals included. And, because all that the Word assumes is saved – that is to say healed – the incarnation saves our animal kin too.

Process theologies such as Keller’s also insist on some kind of panexperentialism thereby maintaining that everything in the world from subatomic matter on up possesses some kind of subjectivity though not conscious self-awareness. In this tradition, ‘matter’ itself is a vast abstraction. Alfred North Whitehead argues that the elementary constituents of reality are actual occasions, events not tiny bits of insensate stuff. Reality is made of self-constituting events which are responsive to all antecedent occasions and ‘the divine lure’.

Process theology posits a thoroughly relational universe, animals and God included. If the rudiments of response-ability were not present all the way down, argue the process theologians, then self-conscious awareness could never have emerged in human and more than human life, at least not without supernatural intervention. Process theologians seek to countermand this kind of arbitrary supernatural intervention whereby the divine instils in one species and one alone, a special supernatural soul substance that alone makes consciousness possible.

This abbreviated litany of constructive theological moves shows just how much preparatory work theologians have already done in order to prepare the theological groundwork for theologies of animality. One way to characterize the overarching goal of theological labour of the varieties I am sketching here is given to us in the aforementioned title of process theologian David Ray Griffin’s still groundbreaking (2000) book, *Reenchantment Without Supernaturalism*. If *pace* Descartes, animals are mere robotic mechanisms, and it is hard to see how taking animals and their lifeworlds seriously is even conceivable. Constructive Christian work seeks to contest reductive materialism, which depicts nature as dead mechanism rather than as living organism; this decisive reframing of theological imagination serves as a vital prerequisite for the possibility of the animal turn.

Arguably, Christian theologians have been at the work of preparing the conditions for the possibility of an animal turn for nearly a century now. To me, Paul, the intriguing question that presents itself is this: what is the relationship between certain kinds of non-supernaturalist Christian enchantments and what you describe as the default orientation of the Pacific Northwest?

## Reverential naturalism and the animal turn

I appreciate your critique of any overly-thick lines one might imagine between religious studies (or the sociology of religion) and theology. In your work – certainly, in *Circling the Elephant* (Thatamanil, 2020) you demonstrate how a theologian might live out the habitus, rules, principles, and values of *Religionswissenschaft.* We are both *homo academicus*, but in my view, you are a rather rare variant. For most discourse about the religion inside and outside of the academy and certainly in the public arena, it is not that difficult to discern the distinctions between as it were theological and secular rhetorics, ways of knowing, authority structures, intellectual objectives, and so forth, and there are good reasons to observe (one might say police) these borders. One approach is not better than the other, and some – again, I would suggest quite exceptional – people are able and happy to cross borders between the two imagined disciplinary countries. The fact that some of us, like you, have dual (or multiple) citizenship does not mean there are not still different rules in place in each country, however. No clever analogy, and no short essay, could clarify these distinctions or settle an always complex debate, but suffice it to say that I take your point and appreciate your critique of an overly stark opposition between these two approaches.^1^

My work on the Pacific Northwest, or the Cascadia bio-region, might shed some light on the concerns that animate this special issue. In *Religion at the Edge: Nature, Spirituality and Secularity in the Pacific Northwest* (Bramadat, 2022; Bramadat et al., 2022*)*, the culmination of a project that involved US and Canadian sociologists, historians, religious studies scholars, religious insiders and non-religious outsiders, we addressed the curious religious landscape of ‘Cascadia’. The region as we defined it includes British Columbia, Washington, and Oregon, with roughly 80% of the population living in an increasingly urban corridor between Portland in the United States and Vancouver in Canada. This bio-region is most often identified with its advanced form of secularization (with the highest level of ‘religious nones’ in North America) (Wilkins-Laflamme, 2018),^2^ the shallow roots of its religious communities, the presence of strong Asian immigration patterns, the ‘you do you’ libertarian ethos, the prominence (in Canada at least) of strong Indigenous revitalization movements articulated in a spiritual rhetoric. Also indicative of Cascadia is its natural splendour, with nature-based tourism being arguably as central a feature of its economy as the mega-corporations based there: Microsoft, Boeing, Lululemon, Intel, Amazon, Nike, and Starbucks, among others.

In the book, I argued that the existing concepts and schemas scholars of religion use to describe and interpret data and social patterns – such as theists, believers, nature religion, eco-spirituality, atheism, adherents, secularization, revitalization, and so forth – seemed inadequate for describing some of what the research team observed. Certainly, conventional forms of Christianity, already less embedded in the formal culture and institutions of the region than elsewhere in North America, have been moving in the same direction, albeit at different speeds and with some exceptions.

However, unsatisfied with calling these people advocates of nature religion, religious naturalism, eco-spirituality, irreligion, atheism, etc., I sought another way to name and frame what I was seeing and hearing (and, truth be told, experiencing myself as a participant-observer-resident). I suggested that what we see in the Pacific Northwest might be called ‘reverential naturalism’.

This is ‘a default orientation, a form of naturalism that accepts scientific approaches to nature and nonetheless frames the natural world in ways that are ‘redolent’ of mysticism, panentheism, and inclusive forms of theism’. It is important to note that I am framing it mainly as a variant of naturalism, rather than a variant of religion. By this, I want to underline that this orientation reflects the end, or the ending, of the taken-for-granted plausibility and power of a Christian paradigm in this region. In this respect, the prominence of reverential naturalism in the region indicates that a threshold has been crossed. Christian privilege is still here, of course, embedded in the social structure of the region; but it is now just one (and arguably a shrinking) element among many.

Since the metaphor has just been articulated, we do not know yet precisely how it might be correlated with social class markers (e.g. income and education level) or important lifestyle orientations (e.g. veganism, recycling, approaches to Indigenous reconciliation). Nor do we know to what extent this perspective is unique to this part of North America (e.g. compared to what one might observe in Iceland). Nonetheless, the concept does appear to name something real and to resonate with people when it is field tested, so to speak. So, while future studies will investigate these correlations, reverential naturalism arguably creates a common ground on which all residents, regardless of formal religious, non-religious, or post-religious identities, can meet. Whether one is a first-generation Portland Muslim, or a Vancouver Millennial three generations removed from any affiliation with Christianity, the sublime features of the natural environment seemed to be part of the taken for granted aspects of life here; it was difficult not to intuit both a tone of reverence from our research participants and a sense of the – here one needs to resort to a now somewhat threadbare metaphor – *spiritual* value of being in relation with the natural world.

I do not want to make a case for the uniqueness of this region or the ways the residents frame the natural world (though in my view it is difficult to beat the beauty and sublimity of the region). I do want to note just how prevalent was the rhetoric of aesthetic uniqueness and reverential naturalism in our engagements with residents. I suspect the reason for this is not just that nature here is more awe-inspiring than elsewhere, but that the state of secularization is so advanced that impressions of the natural world – its meaning, value, and accessibility – develop in a context far more removed from the usual influence of Christian language, ethics, aesthetics, or even a great deal of privilege (and fewer and fewer actual Christians). Indeed, discussions of the natural world that are reliant on categories still moored in traditional Christian metaphors – nature religion, religious naturalism, deep green religion, or even eco-spirituality – seemed inert. This reminds us that reverential naturalism is a default orientation rather than a new ‘religion’, or even ‘eco-spirituality’.

In my view, ‘the animal turn’ we have seen in Christian theology and also in the humanities and social sciences more broadly is quite consistent with the rise of reverential naturalism in the Cascadia bio-region. It is not clear to me, nor is it all that interesting, which came first or whether one is dependent upon the other. It does, however, seem clear that they stand as critiques of an anthropocentric imagination and conventional ways of situating the human as the most important actor in a drama fundamentally oriented to his or her own salvation, separate from the salvation (or love) of the rest of the natural world (not to mention other humans).

It seems that Cascadia’s advanced levels of secularization or deinstitutionalization, create the ideal conditions for the emergence of reverential naturalism as a default orientation to the natural world. This is a laboratory for a post-institutional religiosity that radically decentres both the human animal and the Christian cosmology that justified human dominion over the world’s ‘natural resources’. The region’s reverential naturalism is characterized by its flattening of the hierarchy implicit in the more conventional ways of imagining the supremacy (or even just the primacy) of God vis a vis the world God created. Although I am not an expert in this knowledge, it should be noted that the Indigenous ways of knowing with which I am familiar are arguably fairly consistent with reverential naturalism both because the colonial project arrived later on the West Coast than elsewhere in the continent (so the traditions were perhaps less devastated than they were in Eastern North America) and because these cultural forms tend to avoid binaries between humans and other parts of the natural world.

This is a speculation, but my sense is that the reverential approach to the natural world has become normative here perhaps first in North America because a distinctive kind of secularization is far advanced in the region. As such, there is really no need for a secular orientation to, as it were, overthrow an *ancien régime* (as we witnessed in Quebec during the Quiet Revolution of the 1960s) in order to advance its interests. Instead, the challenge faced acutely today is how to protect the region from rapacious corporations (clear-cutting their ways through old growth forests) and myopic governments (permitting such resource extraction) through rhetoric, narratives, and metaphors that call upon the deeply embedded reverential naturalism.

And in a region in which the foothold of conventional religion is fairly weak, might reverential naturalism permit a distinctive approach to the crises you and I agree threaten all life on this planet? I take it that one of the key tasks of a theologian would be to interpret the reality of Cascadia in a manner consistent with a particular reading of Christian ideals, texts, history, symbols, and hopes. And so, I take it that the irreligious or post-institutional aspect of the region, which is arguably its constitutive feature, might be seen by theologians or clergy as a problem to be solved or a challenge to be met. I am wary of inadvertently countenancing a zero-sum framing of the relationship between ‘the religious’ and ‘the secular’ – but I wonder if in the animal turn and in the prominence of reverential naturalism’s predilection for the notion of an interdependent web of life (as opposed to a more conventional understanding of a creator God standing apart from his creation), some Christians might find themselves no longer on the front lines of an important battle. Of course, I very much appreciate your articulation of the key principles of process theology, though Keller, Whitehead, you, et al, are obviously not articulating the dominant form of Christianity (even among theologians).

You will know I am familiar with the ways leaders in Christian churches see the waning power of institutional Christianity in the region not just as a dilemma, but as an existential crisis for their communities. Many efforts have been made to bring people back to church – better music, evening services, childcare, more inclusivity – and some have been successful. However, it seems to me that there is a kind of acceptance that it is in Cascadia that we see most clearly something like end-state secularization.

However, it seems to me that there are others in your community who approach this moment in the church differently. For them, in the dissipation of what for lack of a better term we can call Imperial Christianity, we see not just the end of a radically anthropocentric and patriarchal Christianity, but an opportunity to move in another direction. For these stalwart Cascadian Christians, perhaps a more prophetic version of the church might emerge that would indeed be more consistent with the movement in its early decades.

## ‘Secular’ and ‘religious’ sources

Paul, I find your ruminations provocative. Read straightforwardly, you offer a neutral and descriptive account of a turn in Cascadia towards reverential naturalism. You give a persuasive account of why it is best described as ‘a variant of naturalism, rather than a variant of religion’. You indicate that this new variety of naturalism may present further challenges to already imperilled Christian institutions. This brings me to a direct response as to whether Christian communities should worry about the reverential naturalist ethos of Cascadia as Paul intimates Christians might. Let me put it plainly: No! What Christians ought to worry about are forms of reductive naturalism that reduce nature to mere matter in motion, naturalisms that reduce nature to mere ‘natural resources’. Akeel Bilgrami (2014) has traced the origins of such reductive naturalism to the form of Newtonianism advanced by the Royal Society which was, he observes, part of a broader set of alliances ‘forged between scientific organizations, commercial interests, and the latitudinarian Anglican establishment’ (Bilgrami: 188–189).

Troublingly for this Anglican, Bilgrami contrasts sharply the reductive naturalism of the Newtonian-Anglican establishment with the ‘enthusiasm’ of the Diggers and Levelers who utterly refused any naturalism devoid of immanent divinity. As Bilgrami tells the tale with great historical nuance, the villains in this narrative are not otherworldly Christians who denigrate nature, but certain sorts of disenchanting Christians who refuse to see nature as anything other than matter in motion. The heroes of the tale are also Christian, namely the radical Christians who refused such crass varieties of naturalism.

This story of Christians against Christians suggests why, like you, I am disinclined to enter into oppositions between secularists/naturalists on the one side and Christians on the other. It matters very much to me what kind of naturalist you are and what kind of Christian you happen to be. Any metaphysic that reduces nature to mechanism that evacuates matter of any sacral qualities, any enchantment, will have in it no room for regarding animals as worthy of ethical regard. Any thoroughgoingly reductive account of nature as mechanism will necessarily regard animals as mere machines. Regardless of label, no variety of naturalism or Christian theology can accord to animality ethical considerability if that naturalism or Christian theology regards nature as a mere matter in motion.

For these reasons, I am unpersuaded that reverential naturalism presents a problem for Christians in Cascadia. The crucial question is whether this variety of naturalism has teeth in it to resist clear-cutting, old-growth logging, and the devastation of animal life supported by these practices.

## Conclusion

Clearly, anthropocentrism is not the particular preserve of a Christian or a sociological imagination. As our conversation has demonstrated, staging such oppositions is inaccurate and unproductive. Why? Because it is possible to be just as anthropocentric with Peter Berger on principles grounded in methodological agnosticism as it would be on certain kinds of Christian theology. Arguably, both sides of this divide have been profoundly conditioned by a Cartesian-Newtonian variety of disenchanted mechanistic naturalism in which animals can only be configured as very sophisticated sorts of automatons, organic robots. It seems to us (both Paul and John) that the question of living well with our animals and others will require extirpation of this particular conceptual kudzu vine, which has invaded both the secular and religious gardens. According to the metaphysics of this variety of naturalism, neither animals nor plants, let alone the inorganic elements of the natural world, have any intrinsic value other than their commercial and commodity value. Although our sciences and our theologies have been corrupted by this invasive species, neither can provide the grounds for living well, let alone living well with our animals and others.

Along the Pacific Coast, the birth of a resident orca in the ‘Salish Sea’ between Seattle and Vancouver makes news. So fragile are their families, and so much does human behaviour threaten their lives, that private and public media outlets understand that residents want to know when one has died or been born. This news is arguably a kind of index for our own vulnerability, living as over 80% of us do in a temperate rainforest constantly threatened by summer fires, close to waters that are likely to inundate us either slowly due to climate change or suddenly due to a massive earthquake that will devastate the region. The beautiful orcas are ‘charismatic mega-fauna’, and emblematic of our hopes and fears; one could say they are the mascots of reverential naturalism.

In the Pacific Northwest of North America, the task of living well together *with all forms of life*, seems somewhat more feasible, if only because while most Cascadians live in cities, the nature of nature here is such that we cannot forget our place within a community of flora and fauna – mushrooms, humans, dogs, cedar, camas, eagles, orcas, oak trees, salmon, cougars, kelp, moss, alder groves, bees, blueberries, etc.

Arguably, for the first time since the European colonization of this region, human residents are bound together and animated by communal forces that have very little to do with capital-R religious ideas, practices, and institutions. The fear in the early decades of settlement was that in the absence of religion or some clear, historically grounded normative system that provided moral certainty and guidance, the broader society would cease to cohere, and all hell would break loose both morally and environmentally. For some Christians, such a fear might still exist, but in our estimation, in Cascadia, actors both religious and post-religious can face the daunting task of responding to the various crises that threaten the region together, with something akin to reverential naturalism, creating a common space in which to experience and think through our shared predicament. Whether one articulates those crises using Christian or non-Christian imagery, the drive to appreciate and protect the space we share seems to bind residents together. Our hope is that this conversation might present an engaging way to think through what our respective fields or disciplines might have to offer the broader society and all of its species.

## Notes

1. I do not want to overstate the differences between our perspectives. I am a serious student and practitioner of yoga, and I suppose I am also a ‘reverential naturalist’. Nonetheless, I try to approach all subjects in equally dispassionate and often ironic ways.

2. Our *Pacific Northwest Social Survey* confirmed how unusual the region is compared to the rest of the continent. The PNSS showed that 49% of British Columbians and 42% of residents of Washington and Oregon had ‘no religion’.
